# Service user perspectives on coercion in mental health

**DOI:** 10.1192/j.eurpsy.2021.97

**Published:** 2021-08-13

**Authors:** A. Nomidou

**Affiliations:** Secretary General, GAMIAN-Europe, Brussels, Belgium

## Abstract

GAMIAN-Europe is a patient-driven pan-European organisation, representing and advocating for the interests and rights of persons with psychosocial disabilities.

While recovery is a deeply personal journey it is also a product of interaction facilitated or impeded through the dynamic interplay of many forces, such as among characteristics of the individual, of the environment and of the exchange. To move recovery forward, recovery-oriented systems in recovery-facilitating environments are needed. Mental health professionals can either facilitate or hinder this journey. Service users and families want to feel they are more than their medical concerns, more than ‘the suicidal’ in room five. Respecting individuals and their human rights, active and engaged listening, including patients in their own healing plan, promoting wellness and engaging with compassion build trust between patients and health care professionals, leading to willingness to follow through with care plans. At the same time, by creating emotional connections and environments, not only can frequent burnouts be prevented, but productivity can be increased.

**Disclosure:**

No significant relationships.

